# Risk factors for bone flap resorption after autologous bone cranioplasty: Protocol for a systematic review and meta-analysis

**DOI:** 10.1097/MD.0000000000021035

**Published:** 2020-07-10

**Authors:** Jingguo Yang, Tong Sun, Yikai Yuan, Xuepei Li, Yicheng Zhou, Junwen Guan

**Affiliations:** a Department of Neurosurgery, West China Hospital; b Health Ministry Key Laboratory of Chronobiology, College of Basic Medicine and Forensic Medicine, Sichuan University, Chengdu, Sichuan province, PR China.

**Keywords:** autologous bone, bone flap resorption, cranioplasty, risk factors

## Abstract

**Background:**

One of the most common complications following autologous cranioplasty is bone flap resorption (BFR). Severe BFR can lead to revision surgery with implantation of synthetic bone flap and also necessarily lead to higher hospital expenses. This study aims to perform a meta-analysis to summarize available evidence regarding risk factors of BFR requiring a second surgery in patients with autologous cranioplasty.

**Methods:**

Cohort, case-control, and cross-sectional studies that report the incidence and risk factors of BFR among patients with autologous cranioplasty, published in English, will be considered for selection. Three databases from inception to May 2020 will be searched. The process of data selection, quality assessment, and data extraction will be assessed by 2 authors independently. The study quality will be assessed by Newcastle-Ottawa Scale (NOS) and Agency for Healthcare Research and Quality checklist.

The statistical analysis of this meta-analysis will be calculated by Review manager version 5.3.

**Results:**

The results of this systematic review and meta-analysis will be disseminated through academic conferences and expected to publish in a peer-reviewed journal

**Conclusion:**

This study will offer high-quality evidence about risk factors for BFR after autologous cranioplasty.

**Registration number:**

INPLASY202050063.

## 1 Introduction

Decompressive craniectomy (DC), a surgical treatment in the management of neurological emergencies, has been demonstrated to reduce mortality rate and improve outcomes for patients with elevated intracranial pressure due to traumatic brain injury (TBI), ischemic stroke, intracerebral hemorrhage, or other causes.^[1–3]^ The procedure is usually followed up by cranioplasty because it is more than a cosmetic repair or restoration of protective barrier of cranial, it can also contribute to the neurological and cognitive improvement.^[4,5]^ Many options exist as to the implanted materials for covering the bone defect, such as autologous bone flap and different synthetic materials.^[6,7]^ Given the biocompatibility and low cost, the autologous cranioplasty is the most common one.

Despite the advantages and simple technique, cranioplasty can carry high rates of complication.^[8,9]^ A frequent and unique long-term complication of autologous cranioplasty is bone flap resorption (BFR).^[10]^ The reported prevalence of BFR with autologous cranioplasty was varied significantly, up to 50% in previous studies.^[11,12]^ BFR can lead to weakening, loosening and significant disintegration of the implanted autologous bone, which eventually results in loss of the bone coverage.^[13]^ Revision surgery with replacement of synthetic material is necessary in severe cases of BFR and second surgery could be associated with higher expenses and poor clinical outcomes.^[14,15]^ It would be reasonable to identify high-risk group that might suffer BFR and take preventive measures or choose alloplastic material cranioplasty for those patients.

The risk factors of BFR, however, remains unclear, and the data is not comprehensive and no systematic review with respect to the prevalence rates and risk factors of BFR has been implemented. Previous reports have found that younger age, bone flap fragmentations, and hydrocephalus shunt implantation to be associated with higher incidence of BFR.^[11,16–18]^ Other potential risk factors, such as bone flap size, preservation of bone flap, and time interval between DC and cranioplasty^[13,17,19,20]^ need to be assessed in a systematic approach. Therefore, we will undertake a systematic review and meta-analysis of studies presented data on risk factors for BFR requiring a second surgery after autologous cranioplasty.

## 2 Methods

### 2.1 Study registration

This systematic review protocol has been registered on the INPLASY website (https://inplasy.com/inplasy-2020-5-0063/) and the study registration number is INPLASY202050063. It is reported to be in line with the meta-analysis of observational studies in epidemiology (MOOSE) guidelines and the Preferred Reporting Items for Systematic Reviews and Meta-analysis Protocol.^[21,22]^ If adjustments are needed throughout the study, we will update the details in the final version.

### 2.2 Dissemination and ethics

The meaning of our findings is to inform the neurosurgeons to take prevention strategies or choose alloplastic materials for high-risk patients with BFR. Hopefully, the results will be disseminated through academic conferences and expected to publish in a peer-reviewed journal. Since this is a systematic review and privacy data is not required, thus no ethical approval is needed.

### 2.3 Inclusion criteria

#### 2.3.1 Type of study

The study will select prospective or retrospective studies (cohort studies, case-control studies) and cross-sectional studies will also be included. The language of literature will be limited in English, but there will be no restriction on publication data. Case report, letters, conference abstracts, reviews, non-clinical research, technical note will be excluded.

#### 2.3.2 Participants

In study group, any patients should be diagnosed with BFR requiring a second surgery after autologous cranioplasty with no restrictions on ethnicity, sex, or nation. People in control group should be patients without BFR or those patients with BFR but not requiring a revision surgery with implantation of synthetic materials. The diagnosis of BFR will be based on valid clinical and radiographic findings.

#### 2.3.3 Outcomes

The outcomes should be explicitly reported as the followings:

1. Incidence of BFR;
2. Study reported at least 1 risk factor for BFR requiring a second surgery;
3. Study reported findings in terms of risk estimate (odds ratio, relative risks, hazard ratio) or provided sufficient data to calculate. Those studies that risk estimates cannot be directly extracted or obtained will be excluded.

### 2.4 Search strategy

A systematic and comprehensive search will be carried out in 3 databases, including PubMed, Embase, Cochrane Library database, from the inception to May 1, 2020. Detailed search strategy of PubMed will be ((((cranioplasty[Title/Abstract]) OR post-cranioplasty[Title/Abstract])) AND (((((autologous[Title/Abstract]) OR bone[Title/Abstract]) OR autologous bone[Title/Abstract]) OR autogenous[Title/Abstract]) OR autograft[Title/Abstract])) AND ((((resorption[Title/Abstract]) OR necrosis[Title/Abstract]) OR bone resorption[Title/Abstract]) OR bone necrosis) The search strategies for other electronic databases will be modified appropriately.

### 2.5 Study selection

**Figure 1:**
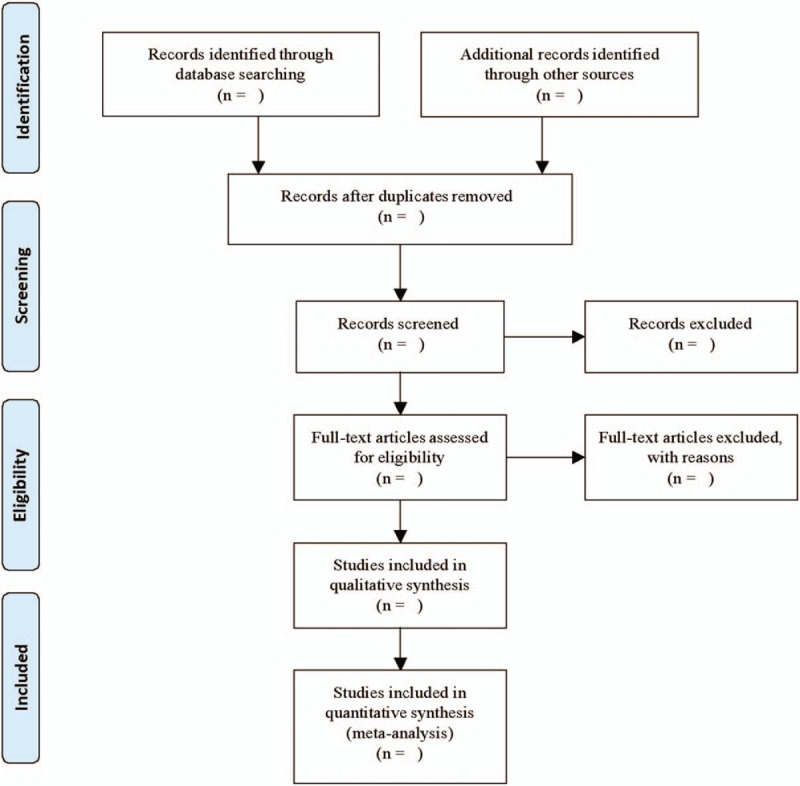
Study selection flow diagram.

Two independent members in our group will select all of the studies and import them into Endnote version X8 software to manage. First, the duplicated studies will be removed. Then, the 2 authors will independently screen all potentially qualified studies by reading titles and abstracts. Studies will be excluded if they do not meet inclusion criteria. Finally, the 2 authors will screen the full text and determine the final qualified articles that in line with the inclusion and exclusion criteria. The 2 reviewers will crosscheck the included studies, disagreements in the process will be resolved after mutual discussion. If no agreement is reached, the third author will individually evaluate the matter. The Preferred Reporting Items for Systematic Reviews and Meta-Analyses Flowchart (Fig. 1) will be filled to provide specific information.

### 2.6 Data extraction

For eligible studies, data were independently extracted into Microsoft Excel by 2 authors. The following information were collected: name of the first author, study period, preservation of the bone flap, where the study was conducted, study design, sample size, mean (median) age of participants, the rate of bone flap resorption. We will extract the risk factors if they reach statistical significance of 5% in univariate and multivariate analyses. The risk estimates with a 95% confidence interval (CI) were extracted for each risk factor or the absolute number of case and control for each risk factor were available. If data is missing, we will email the corresponding author to ask for assistance.

### 2.7 Quality assessment

All included studies will be cohort, case-control and cross-sectional studies. The quality of included studies will be independently assessed by 2 authors and possible discrepancies will be adjusted by a third author. Quality assessment will be conducted according to guidance of Newcastle-Ottawa Scale (NOS),^[23]^ which contains 3 categories: 4 items for patients selection, 1 item for study comparability and 3 items for outcomes assessment. The score is classified into 3 scales: 7-9 defined as good, 5-6 is fair quality, and 0-4 is poor quality.

### 2.8 Data analysis

#### 2.8.1 Meta-analysis

Meta-analysis will be performed using Review manager version 5.3. The outcome measures for the meta-analysis will be risk factors associated with BFR requiring revision surgery. If relevant risk factors are reported in 2 or more studies, the pooled odds ratio with 95% confidence intervals will be calculated. If the risk factors could not be included in this meta or reported only once, the results will be presented separately or in discussion part.

#### 2.8.2 Measures for heterogeneity

Heterogeneity of the studies will be assessed using Cochrane Q test and *I*^*2*^ index. When *P* value <.10 or the value of *I*^*2*^ < 50%, studies will not be considered heterogeneous and a fixed effect model will be adopted in the meta-analysis; otherwise, a random effects model will be applied.^[24]^ In the case of heterogeneity, the quantitative synthesis is not appropriate, the results will be presented in tables or charts.

#### 2.8.3 Subgroup analysis

If the results of meta analyses are heterogenous, we will perform a subgroup analysis based on several aspects, such as race, study country, study year, different bone flap preservation, and study quality.

#### 2.8.4 Sensitivity analysis

The sensitivity analysis is used to evaluate the robustness and stability of conclusions. It will be conducted by removing low-level quality study one by one and then merges the data to probe the sample size, study quality, and missing data on results of the study.

## 3 Discussion

BFR is one of the most common complication following autologous cranioplasty, which could lead to prolonged hospital stay and neurological deterioration and economic burden. Recently, there has been a growing number of studies about identification of risk factors and potential strategies for lowering BFR rates, avoiding a second surgery. The results are different and no studies have summarized the existing evidence. Therefore, the purpose of this systematic review and meta-analysis is to summarize the evidence from previous researches and investigate potential risk factors for BFR. For those patients with risk factors, prevention strategies or implantation with alloplastic material are necessary.

There are strengths in this study. This is the first meta-analysis to summarize findings about risk factors for BFR, which could provide clear evidence for clinical work and improve clinical outcomes. However, there may be some limitations in our meta-analysis. First, we only search 3 international database that may lead to selection bias. Second, the included types of studies are varied, for example RCTs, case control studies and cohort studies, this may cause substantial heterogeneity. Third, the methods of bone flap preservation are different, this may also be a source of heterogeneity.

In conclusion, this study will help to identify risk factors for BFR after autologous cranioplasty. We hope this systematic review and meta-analysis can provide a high evidence for predictions for BFR and guide future clinical works.

## Author contributions

**Conceptualization:** Jingguo Yang, Junwen Guan.

**Data curation:** Jingguo Yang, Yikai Yuan, Yicheng Zhou.

**Methodology:** Tong Sun.

**Project administration:** Xuepei Li.

**Writing – original draft:** Jingguo Yang.

**Writing – review & editing:** Junwen Guan.
